# Can Carbon Quantum Dots (CQDs) or Boron Compounds be an Ultimate Solution for COVID-19 Therapy?

**DOI:** 10.22037/ijpr.2021.114856.15071

**Published:** 2021

**Authors:** Gamze Camlik, Esra Kupeli Akkol, Zelihagul Degim, Ismail Tuncer Degim

**Affiliations:** a *Department of Pharmaceutical Technology, Faculty of Pharmacy, Biruni University, 34010, Topkapı, İstanbul, Turkey. *; b *Department of Pharmacognosy, Faculty of Pharmacy, Gazi University, 06330, Ankara, Turkey.*

**Keywords:** COVID-19, Boron, Nanocarriers, Quantum dots, Pandemic

## Abstract

Severe acute respiratory syndrome (SARS) is an infectious and highly transmissible disease that is affected by SARS coronavirus (SARS-CoV) and for which there are presently no approved treatments. COVID-19 is a new strain of coronavirus that has not been previously identified in humans. It is also a member of the *coronaviruse* family and known to cause similar illnesses in humans. The last outbreak has been identified as a Pandemic because of COVID-19 infections in humans. This review has been prepared to give some information to readers or scientists about some new generation of boron-doped or boron attached composite quantum dots during the design phase of the drug or drug delivery systems to be developed to combat COVID-19 and to help in the design of new drugs and systems by opening some new horizons. All scientists and researchers must quickly share their ideas and experiences in the fight against COVID-19 to find a better therapy or strategy for humans, and thus we can be successful. In this sense, this review offers readers some new ideas and rational perspectives. In conclusion, boron-containing composite carbon quantum dots appear to be the most suitable delivery system for treating COVID-19 infections especially when they are delivered through the lung.

## Introduction

People have been struggling to survive ever since they were born, and it took the time and effort to fight diseases and germs the most. Many diseases have threatened people’s lives throughout history, and even at certain times, many people have died because of many different pandemics. Many microorganisms have caused diseases from time to time, and there have been severe deaths. Some drugs, vaccines, and even treatment strategies have been developed and used, but there is still a general need to develop all these more. The more efficient therapy and treatment strategies, the more life qualities and life expectancies or lesser treatment expenses can be obtained. One of the biggest disasters that happened in the world in recent years is the COVID-19 outbreak. COVID-19 is a new strain of coronavirus that has not been previously identified in humans. The COVID-19 is also a member of the coronaviruses family is known to cause illnesses ranging from the common cold to more severe diseases such as Severe Acute Respiratory Syndrome (SARS) and Middle East Respiratory Syndrome (MERS). Fighting this disease and its causative virus is quite difficult because it is very contagious and does not have a fully effective drug that can be given systemically other than the respiratory route. In this case, the most rational approach is to develop the possibilities we have, in other words, to combat this virus with the possibilities we have. In fact, if we carefully evaluate the COVID-19 virus, we can see that it settles in the lungs and causes death by causing damage there. Therefore, a good idea may be to try blocking the virus settling in the lungs as a starting point. The other important issue is the size of the virus. It is reported to be about 120 nm (1). It will not be wrong to think that if such a small virus is to be dealt with, the drug delivery system should also be of this size or smaller. At this point, quantum dots appear in our scientific scene to be discovered and used in COVID-19 treatment. However, many known quantum dots (CdSe or CdTe) are small (1-10 nm), highly toxic due to inherently containing cadmium (2), and it is wiser to use them in a short period of time at low doses in diagnosis rather than as a drug or drug carrier. However, the introduction of new generation non-toxic, composite carbon quantum dots and their potentials against viruses or as a drug or delivery systems are presented in this review. Besides, some of the boron’s superior properties are given in this review.

COVID-19 infections

Coronaviruses (CoV) are members of a large family of viruses, and in general, cause quite severe infections in the community, such as the common cold, self-limiting mild infection symptoms, MERS or SARS (3).

There are several subtypes of coronaviruses (HCoV-229E, HCoV-OC43, HCoV-NL63, and HKU1-CoV) found in humans, and they can easily be transmitted from one person to person. These virus subspecies that circulate among humans can cause colds. However, there are many coronavirus subspecies in animals, and these can be passed to humans and cause severe disease. As a result of detailed research, it has been revealed that SARS-CoV’s can be transmitted to humans from musk cats, and MERS-CoV can be transmitted from single-humped camels (4).

SARS-CoV emerged as a previously unknown virus but in 2003 as the first international health emergency case of the 21st century, causing hundreds of people to die. MERS-CoV, which has not been shown in humans or animals before. Ten years later, the first coronavirus was described in humans in Saudi Arabia in September 2012. However, it was later revealed that the first cases were actually in Zarqa, Jordan (4).

On December 31, 2019, the WHO in China Country Office reported some pneumonia cases with an unknown etiology in Wuhan, China’s Hubei province. On January 7, 2020, the causative virus was identified as a new coronavirus (2019-nCoV) that was not previously detected in humans. Later, the name of the 2019-nCoV disease was accepted as COVID-19, and the virus was named SARS-CoV-2 due to its similarity to the SARS-CoV (5-7).

The WHO categorized the COVID-19 epidemic as an international public health emergency on January 30. The global epidemic (pandemic) was announced on March 11 due to the incidence and severity of diseases in 113 countries. The first outbreak was started in China (8).

The virus that causes COVID-19 is mainly transmitted through droplets generated when an infected person coughs, sneezes, or exhales. These droplets are too heavy to hang in the air and quickly fall on floors or surfaces. Any person can get infected by breathing -containing air or if they are close to someone with COVID-19. It is also possible to get contaminated by touching contaminated surfaces and then touching the eyes, nose, or mouth (9).

Many articles deal with treatment difficulties, side effects, potentially detrimental, and irreversible complications for COVID patients (10). Although personal hygiene has a crucial role in preventing an infection from others and washing hands with soap frequently or using hand sanitizers are the basic preventive measures COVID-19 is still a very contagious disease (11). Moreover, this disease is a disease that is very difficult to treat once the infection has started and is very contagious. Severe acute respiratory syndrome (SARS) is a respiratory disease that occurs with the COVID-19 virus, and the disease that has spread to many countries has affected many people (12, 13). COVID-19 is most often transmitted through the respiratory tract (14, 15). People who have the infection became ill after short days of incubation. Many of these patients possibly will be in a critical stage. They will probably need to be in intensive care. This can pose a major challenge for limited hospital resources and capacities in the case of a sudden spike in infections. The biggest problem encountered in the treatment is that there is no effective drug or vaccine for a complete or definitive solution.

*Strategy for the therapy and rationale*

Currently, only a few drugs are officially available to treat COVID. Ribavirin was initially used, but it turned out to be an almost ineffective drug and shows serious side effects (16, 17). Interferon was initially reported to exhibit an anti-COVID-19 effect (18), and glycyrrhizin was also found to be effective (19), but then the hope for them lost rapidly. Although some antimalarial drugs such as hydroxychloroquine or some antiviral drugs like favipiravir or remdesivir are recommended, the exact effect has not been revealed. For instance, remdesivir is an antiviral drug targeting a range of viruses. In fact, it was originally developed to treat hepatitis C and a cold-like virus called respiratory syncytial virus (RSV) over a decade ago. Remdesivir was not effective on COVID-19 disease, but it may affect the other viruses. According to the recent NIH reports, the latest findings show that remdesivir alone cannot provide sufficient treatment for COVID-19 infected patients but does provide some benefit (20). 

At the same time, some drugs that inhibit the entry of the virus into the cell have been developed, for example, a new class of virus entry inhibitors has been developed for human immunodeficiency virus (HIV) (21, 22), and one of them, enfuvirtide, is now being tested in the clinic (23, 24). For the respiratory syncytial virus (RSV) entry blockage, small molecules coded RFI 641 (25, 26) and VP-14637 (27) were developed. Entry inhibitors, signaling peptides (28, 29), *n*-docosanol (30), and FGF4 for the herpes simplex viruses are still developing. There are few attempts to use the repurposed drugs, but none is good. In short, there is still a general need that the more effective treatment and the more potent drug and delivery system should be found.

*Finding an effective drug or a better delivery of the known drug*

The real problem starts right here. If we consider all the processes for drug discovery or development for a new formulation or licensing processes, we do not have enough time to complete all these processes, and exploring or finding a new drug may not be possible within a short time. A recent report published by the Tufts Center for the Study of Drug Development (TCSDD) pegs that the cost of developing a prescription drug that gains market approval can be around $2.6 billion considering an increase in inflation rate in 2003. TCSDD’s finding, a bellwether figure in the drug industry, is based on an average out-of-pocket cost of $1.4 billion and an estimate of $1.2 billion in returns that investors forego on that money during the 10-plus years a drug candidate spends in development (31). The center’s analysis drew from the information provided by 10 pharmaceutical companies on 106 randomly selected drugs first tested in humans between 1995 and 2007. This study concludes that another $312 million is spent on post-approval development—studies to test new indications, formulations, and dosage strengths—for a life-cycle cost of $2.9 billion. The virus is always ahead of us. Therefore, some scientists say that an effective vaccine should be developed. However, biological variations between the immune system and humans may not allow us to do this. For this reason, we should focus our attention in a way that rather rapid results and make an effective war with the weapons we already have. What we need to consider here is that to determine what potential and effective strategy we have. At this point, we can realize that we focus on an erroneous strategy for effective therapy. It is an important point that choosing the correct path and the target. It may perhaps not a correct approach to give the drug orally or systematically and expecting it to reach an adequate concentration in the lung. Because, in this case, both higher doses have to be used and it should wait a long time for the drug to reach a sufficient concentration in the affected area. Meanwhile, the virus continues to work and be harmful. Moreover, hypersensitivity reactions and cytokine storm syndrome can carry the treatment to very negative points due to overdose. At this point, local targeting with a lower dose may be a good solution. Administration of drugs by inhalation can be an important advantage here.

At this point, we need to develop a new drug delivery system. As a matter of fact, this disease is a lung disease. Most of the deaths occur due to the impaired function of the lung. In other words, the target organ for effective therapy should be the lung. Orally or systemically given drugs can reach a sufficient concentration in the lung after a specific time. The virus infection spreads rapidly and invades the lung cells in a short time. Sudden death can happen. There is no time to waste and jeopardize the health of patients. For this reason, it would be pretty advantageous if that delivery system that can be delivered the drug directly to the lung. Therefore, the basic strategy should be to use a drug delivery system targeted to the lung. However, this cannot be like asthma and carrier systems in COLD (Chronic Obstructive Lung Diseases) or conventional inhalation products used in asthma or solid lactose particles because the particle sizes of the systems or carriers (like lactose) are around 1 to 5 micrometers (32-34). These particle sizes are not suitable to deliver the drug to the deeper part of the lung. However, COVID-19 infection involves the lung epithelium at the alveolar level. For this reason, the droplet or the particle size of the carrier system should be much smaller. Our current knowledge can make us think that if the particles delivered to the lung are too small, they are more likely to be expelled or cleared by an exhalation. Current reports indicate that smaller particles can reach the alveoli (32-34). So, the system should have an optimum particle size, which can be monitored, easily passed through cell membranes, or it can transport the drug to this target area perfectly. At this point, quantum dots appear as a very suitable carrier nanoparticular system. 

*Quantum dots*

**Figure 1:**
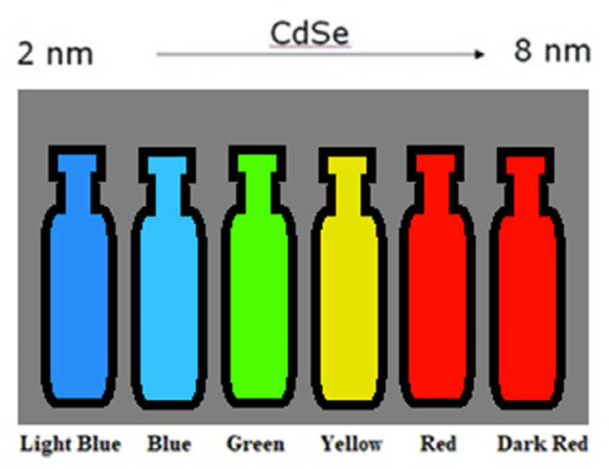
Size and color dependencies of QDs

Quantum dos (QDs) are like a tiny crystal cage with a few to thousands of atoms. Although they can accommodate up to thousands of atoms, most of the synthesized QDs are usually within the size of 2-15 nanometers (10-75 atoms). QDs can be obtained from the compounds of the periodic table II-VI, III-V group. It is possible to obtain QDs from almost all semiconductor-metal compounds. QDs have been produced from CdSe, InAs, CdS, GaN, InGeAS, CdTe, PbS, PbSe, ZnS, and ZnO. The reason they are called QDs is that the bandgap can be changed by changing its dimensions. In other words, size is a controllable parameter in QDs, and when this feature is combined with the effect of quantum confinement, quantum dots can gain some extraordinary optical and electrical properties (35). Because with the change of the dimensions of the QDs, the color of the emitted light changes with the effect of quantum restriction. While the smallest size QDs look blue, large QDs emit red light (36) (Figure 1).

As a result, it is a controllable process to excite and make them emit light at all frequencies which can be seen with QDs, and even it is possible to get infrared emission. In this way, QDs have a great potential for medical therapies, imaging processes, LEDs, solar panels, electronic and computer applications. QDs have been started using extensively in medicine or pharmacy, especially in recent research, in the adequate transportation of drugs, their targeting, and especially in the treatment of cancer (37).

*Safety concerns with QDs*

Quantum dots generally have a very toxic effect since they are made up of metal atoms. Even though the quantum point, which has very superior properties, is generally made, their toxicity could not be reduced, which is a significant problem. The ultimate effect and the real biodistributions or changes of biodistributions of QDs in the body when some conditions change are not fully understood and determined yet. At this stage, carbon quantum dots (CQDs) appear to be the safest nanoparticles to be used for medical purposes. In recent years the production and preparation methods of CQDs have been proposed. The very futuristic usage of CQDs has been shown in the literature. Carbon materials, including CQDs are generally accepted as safe to be used. Carbon materials are also adsorptive material, which is useful for delivering the drug molecule by simple adsorption and subsequent desorption. If the carbon material is good enough to carry, the drug molecule can be released at the site of action by desorption. It is a rather easy procedure and the material is known to be safe. The only problem is the size of the carbon material because many times the material is safe when it is in bigger form but it can be very toxic if it is in nanometer size. Besides many other particulate products, nanoparticles (NPs) have been widely used in diverse food fields, including food processing, safety assessment, packaging, and nutrition delivery (38, 39). These NPs may potentially enter the body via several different routes of inhalation, ingestion, or uptake through the skin (40). All these show some nanoparticles may be safe. It is very interesting that when NPs are exposed to biological fluids or when they enter the body, they will probably be covered immediately with the protein or they are claimed to be covered and form a kind of protein coronas on the nanoparticles (41, 42). This indicates the affinity of nanoparticles to protein. If NPs are smaller and this version of nanoparticles can be QDs. The attraction of protein to the QDs maybe even more. The interaction between nanoparticles and protein also affects the toxicity (43, 44). The formation of the protein nanoparticle complex depends on size, chemical composition, and surface characteristics (45). S Hu *et al.* reported that carbon material (graphene oxide) can form a complex with 10% fetal bovine serum, thus mitigating the cytotoxicity (46). Coating graphene oxide with bovine serum albumin significantly attenuated its toxicity.

The food-borne CQDs have been found in roast salmon after the flesh of fish was heated at about 200 ^o^C for 50 min (47). When roast salmon is consumed, the CQDs are inevitably transferred into the circulatory system. It is possible to get CQDs when we eat roasted salmon. These CQDs may not be very dangerous. These food-borne CQDs might encounter various kinds of serum proteins and be absorbed by these proteins via interactions of the functional groups (48). Among the serum proteins, human serum albumin (HSA) is the principal soluble protein constituent (40 mg/mL) in human blood plasma with many physiological functions (49) and it has been shown that CQDs can really interact with proteins in the body45. The formation of the human serum albumin (HSA) and CQDs complex like a corona from roast salmon and biological effects, including acute toxicity in mice have been investigated. The HSA-CQD complex has been introduced because of the static binding mechanism (50). The HSA-CQD complex is mentioned to have entered the cytoplasm and they were found to be present in lysosomes or autolysosomes. The HSA coronas reported to mitigate the cytotoxicity of CQDs from 18.65% to 9.26%, and the energy metabolism was rectified from glycolytic to aerobic metabolism (51). This shows the detoxification mechanism and the affinity of proteins to the CQDs. The COVID virus has proteins (S1 and S2) to bind to the receptor to enter the cell. It has been reported that if S proteins are blocked with a molecule it can be used for preventing the host cells from COVID entering (51). If CQDs are not very toxic and if we still consume them without noticing and having no toxicity problem, they may be useful for preventing COVID infections. Because CQDs are capable of interacting with proteins and the coronavirus has a protein to enter the cell; we may be able to stop the virus using CQDs. It was indeed a very interesting study that showed QDs made from tea leaves lay waste to lung cancer cells (52). Their research confirmed previous evidence that tea leaf extract can be a non-toxic alternative to making QDs using chemicals. The cadmium sulfur (CdS) quantum dots derived from tea leaf extract reported to show exceptional fluorescence emission in cancer cell bioimaging compared to conventional CdS nanoparticles (52) but Cd is still not very good to use being quite toxic even carcinogenic element. However, all these show us quantum dots can be a good alternative for the therapy of lung diseases reminding COVID-19.

At the same time, there is another fascinating paper appearing in the literature highlighting the positive effect of functional CQDs as medical countermeasures to human coronavirus (53). Researchers produced a series of functionalized CQDs. They tested their functionalized CQDs in terms of antiviral activity. It was very interesting that all boron functionalized CQDs were found to be antiviral. Moreover, when they functionalized their CQDs with amino boronic acid they found that the antiviral effect was maximum (EC50 = 5.7 mcg/mL). Authors claim that the underlying mechanism of action of these CQDs can be revealed to be inhibition of virus entry that could be due to the interaction of functional groups of the CQDs with virus entry receptors inhibition; the activity was reported to observe at the viral replication step (53). If the boron-containing CDQs are effective for the therapy of COVID-19, boron compounds or boron doped CQDs may be a better alternative.

*Boron compounds and composite CDQs (CCQDs): Boron doped CQDs*

For years, boron and its compounds have been used in the medical and pharmacy area for antiseptic or antiviral purposes. The safety aspect of the use of boron or boric acid was tested long ago (54). Boron containing water-emulsifying ointments for napkin area hygiene has been investigated and reported that lack of absorption was observed from the napkin area of babies (55), lack of absorption from was found even abraded skin areas of adults and very low *in-vitro* releases of boric acid to a dialysis system from such ointments have observed (56). The latter investigation also demonstrated a nearly quantitative elimination of an oral dose of boric acid either in aqueous solution or in a 3% water-emulsifying ointment in man over 96 h. and precise renal clearance determinations have been performed (57). The full pharmacokinetics of this compound has been determined (58). All these show that boron compounds can be excreted by urine and at doses, they are not very toxic. It is quite interesting that although boron offers a rare fantastic opportunity to design or explore and pioneer its utility in chemotherapeutics, boron has not been studied well, and in general it has not overlooked. The main reason for that is possibly a wrong common belief and it may be hard to get boron compounds easily to work with. In many countries, boric acid is known to be an ingredient of insect poisons. However, it has to be largely unfounded because the LD50 value of boric acid is around 2.7 g/kg where table salt LD50 is 3g/kg in rats by the oral route (59). Moreover, boric acid has been used as a preservative in eyewashes or vaginal preparations and boron is also present in many fruits, vegetables, and some nuts at quite high concentrations. It has been found that we consume 0.3 to 4.2 mg of boron every day (60). All these show that boron is not an inherently toxic elements like mercury and boron can be tolerated quite well. In boron neutron capture therapy (BNCT) 100 mg/kg boron has been administered to the patients proving that it is safe (61).

*Boron as a useful atom*

Boron is a simple and useful atom. Boron has a wide range of applications in drug discovery chemistry, materials science, energy research, electronics, and the life sciences. The chemistry of boron provides many opportunities. One of the reasons for this is its location in the periodic table of elements and its electronic structure. Boron places next to carbon in the periodic table, which causes boron to share some similarities with carbon but also possesses some important properties. The combination of these similarities and differences gives boron its potential in drug discovery, design, and medicinal applications. In contrast to single boron atom compounds, boron atoms in boron clusters act in concert to create new qualities such as three-dimensional structures, aromaticity, hydrophobicity, and the formation of non-classical proton-hydride bonds. All make the boron atom unique (62). Boron can be reckoned as a similar but opposite atom of nitrogen that possesses a wide range of opportunities. Nitrogen is a Lewis base, but boron is a Lewis acid; boron has an empty p-orbital in which nitrogen has a full p-orbital (lone pair). Boron is electrophilic; where nitrogen is nucleophilic. Moreover, the empty p orbital can be occupied by a lone pair meaning that boron can form a dative bond with nucleophiles like nitrogen of enzymes, proteins,* etc.* (63). 

**Figure 2:**
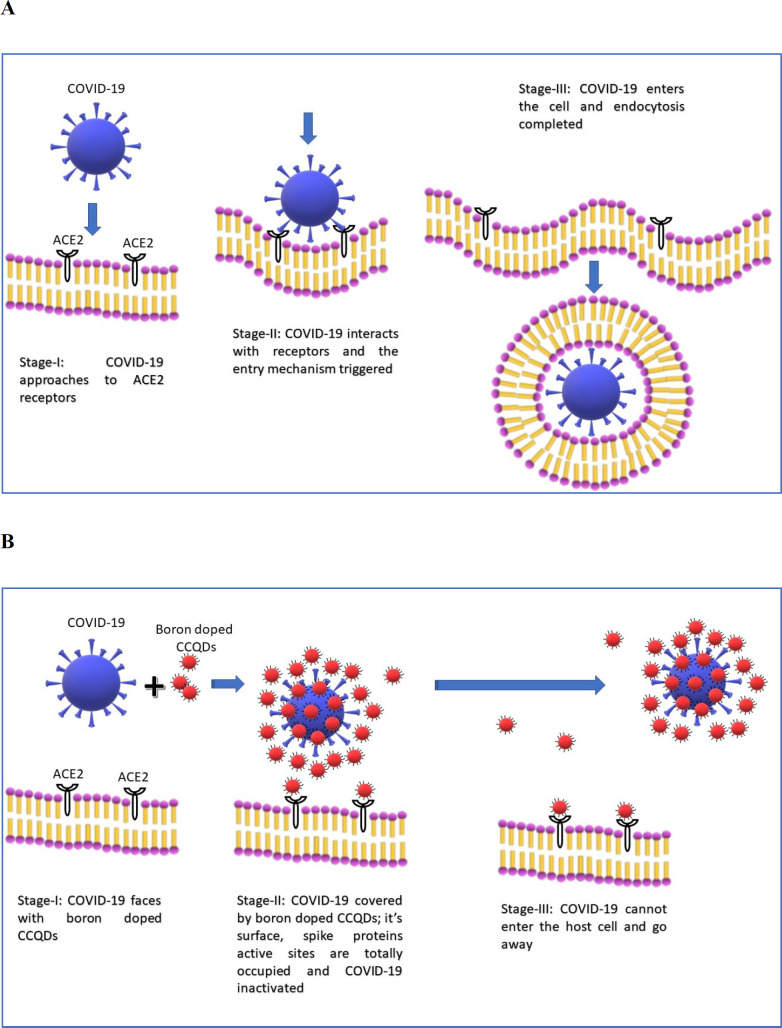
The proposed antiviral mechanism obtained with boron doped CCQDs. This figure shows the blockage of COVID-19 viruses at the lung, at the entrance, at the cell surface. Blocking the viruses with boron doped CCQD results in no entrance when CCQDs were administered to the lung. (A) Normal circumstances; (B) When Boron doped CCQDs were administered to the lung

There are some articles about boron derivatives and their antiviral activities in the literature. One of them published in Russia related to the antiviral effect of boron-adamantane complexes (64). Previously mentioned important research with functionalized CQDs with amino boronic acid decoration was found to have an antiviral effect because of boron and related group affinity to the amino group of S2 protein (53). This virus blocking effect can be seen because of that. The thing is that if we are going to fight this virus with the size of 100 to 120 nm (1), the pharmaceutical system should be suitable for this. At this stage, quantum dots having boron atoms appeared to be very good in size, and their functions and affinities to coronaviruses look quite acceptable. Boron has an antiviral effect in many forms, one literature mentions all these boron-containing compounds and their antiviral activity. Figure 2 summarizes the activity of boron doped CCQDs antiviral activity. The attraction between boron and amine groups makes a strong bond-like interaction and this protects virus entry by both blocking S2 protein binding and viral replication (53). 

Administration way of drug formulation

COVID-19 target is lung epithelial cells therefore, our target for stopping viral entry should be the epithelial surface of the lung. If it is the case, we can deliver the drug formulation using an inhaler; drug formulation can be sprayed and delivered by inhalation. In this case, the drug formulation should be a solution, or dry powder can also be possible. The solution may be a bit better because, if a powder form of CCQDs is going to be used; the actual particle size will be quite small; in this case, particles can be reached to the deeper site of the lung; even maybe to alveoli, but they can be exhaled as well. If the sprayed form is applied to the lung by inhalation, the droplet size can be controlled better using a good spray head and pressure. Therefore, it can be sent to the site of action much better. When CCQDs reach the surface of the epithelial cells in alveoli, they can interact with COVID-19 viruses, block the adhesion, or even stop viral replication.

As mentioned earlier, CQDs are a new class of fluorescent carbon nanomaterials with an approximate size in the range of 2–10 nm. The majority of the reported review articles have discussed the development of the CQDs, especially for use in bio-imaging and chemical-/biological-sensing. However, there is still a severe lack of consolidated knowledge on the recently developed CQDs (especially doped/co-doped) and their therapeutic effects. However, there are a number of studies present in the literature indicating some recent developments in doped and co-doped CQDs using boron (B), fluorine (F), nitrogen (N), sulfur (S), and phosphorous (P) (65). The green synthesis methods of these boron-doped CCQDs has been also introduced (66) but many extraordinary properties of these CCQDs still need to be discovered.

Hurdles and limitations for CCQDs for COVID-19 therapy

Since the discovery of CQDs, several simple, low-cost, and efficient routes for the synthesis of CCQDs have been developed. This is in drastic contrast to the other expensive chemistry required to syntheses and for other fluorescent nanoparticles and the other QDs. The main hurdles during its synthesis are the reproducible physicochemical properties of CCQDs, their fluorescence properties, including photobleaching and photo-blinking, and chemical stability (67). Unfortunately, the other problem may be obtaining high quantum yields, which remain rare. Although significant findings have been reported concerning the applications of CQDs, their exact mechanism of cellular uptake and precise toxicological effect remain to be explained since the pharmacokinetics and bio-distribution of CCQDs are dependent on many known and unknown factors. CCQDs are so small and non-specific capturing can be seen by macrophages, which can also affect their excretion route, circulation half-life, and these are still not fully determined yet (67). Cytotoxic effects of CCQDs have been waiting to be determined in detail. The development of CCQDs for drug delivery for human use is still in the midst; CQDs have already shown immense potential to play a big role in nanotechnology for the development of assays, sensors, bioimaging agents, drug carriers. Many optical and electronic properties of CCQDs and the biological effect after long-term usage are not well understood yet.

## Conclusion

All these theoretical aspects, experimental results show that boron-doped CCQDs may be a better drug or delivery system for effective and rational therapy alternatives for life-threatening diseases, including COVID-19 infections. Especially, boron attached or boron-doped CCQDs appeared to be the most effective one. Delivering the drug with a delivery system to the lung in a spray form may be another alternative because the target is the epithelial surfaces of lung cells. The boron doped CCQDs (as a delivery system or a drug can simply be administered by inhalation of its sprayed solution. This may be a better strategy to stop viruses at the entering site. CCQDs may be a starting material to be developed more for various purposes. We strongly believe that these results and points of view can help readers to think through a new pathway. This may open a new window to fight other diseases with new or better strategies.

## Author Contributions

 “Conceptualization, I.T.D.; collection of literatures, G.C, E.K.A., and Z.D.; review writing, study design, G.C., E.K.A., Z.D., and I.T.D.; editing, G.C., E.K.A.,Z.D., and I.T.D.

## Funding

This research received no external funding.

## Conflicts of Interest

The authors declare no conflict of interest. 
